# Using cryoprobes of different sizes combined with cone-beam computed tomography-derived augmented fluoroscopy and endobronchial ultrasound to diagnose peripheral pulmonary lesions: a propensity-matched study

**DOI:** 10.1186/s12931-024-02700-w

**Published:** 2024-02-05

**Authors:** Ching-Kai Lin, Sheng-Yuan Ruan, Hung-Jen Fan, Hao-Chun Chang, Yen-Ting Lin, Chao-Chi Ho

**Affiliations:** 1 Department of Medicine, National Taiwan University Cancer Center, Taipei, 106 Taiwan; 2 Department of Internal Medicine, National Taiwan University Hospital, 7, Chung-Shan South Road, Taipei, 100 Taiwan; 3 Department of Internal Medicine, National Taiwan University Hsin-Chu Hospital, Hsin-Chu 300, Taipei, Taiwan; 4 Institute of Epidemiology and Preventive Medicine, National Taiwan University, No.17 Xu-Zhou Road, Taipei, 10020 Taiwan; 5 Department of Internal Medicine, National Taiwan University Biomedical Park Hospital, Hsin-Chu County 302, Taipei, Taiwan

**Keywords:** Cone-beam computed tomography-derived augmented fluoroscopy, Cryoprobe, Diagnostic accuracy, Endobronchial ultrasound-guided transbronchial biopsy, Peripheral pulmonary lesions, Transbronchial cryobiopsy

## Abstract

**Background:**

Endobronchial ultrasound (EBUS) and cone-beam computed tomography-derived augmented fluoroscopy (CBCT-AF) are utilized for the diagnosis of peripheral pulmonary lesions (PPLs). Combining them with transbronchial cryobiopsy (TBC) can provide sufficient tissue for genetic analysis. However, cryoprobes of different sizes have varying degrees of flexibility, which can affect their ability to access the target bronchus and potentially impact the accuracy. The aim of this study was to compare the diagnostic efficacy of cryoprobes of varying sizes in CBCT-AF and EBUS for the diagnosis of PPLs.

**Methods:**

Patients who underwent endobronchial ultrasound-guided transbronchial biopsy (EBUS-TBB) and TBC combined with CBCT-AF for PPLs diagnosis between January 2021 and May 2022 were included. Propensity score matching and competing-risks regression were utilized for data analysis. Primary outcome was the diagnostic accuracy of TBC.

**Results:**

A total of 284 patients underwent TBC, with 172 using a 1.7-mm cryoprobe (1.7 group) and 112 using a 1.1-mm cryoprobe (1.1 group). Finally, we included 99 paired patients following propensity score matching. The diagnostic accuracy of TBC was higher in the 1.1 group (80.8% vs. 69.7%, *P* = 0.050), with a similar rate of complications. Subgroup analysis also revealed that the 1.1 group had better accuracy when PPLs were located in the upper lobe (85.2% vs. 66.1%, *P* = 0.020), when PPLs were smaller than 20 mm (78.8% vs. 48.8%, *P* = 0.008), and when intra-procedural CBCT was needed to be used (79.5% vs. 42.3%, *P* = 0.001). TBC obtained larger specimens than TBB in both groups. There is still a trend of larger sample size obtained in the 1.7 group, but there is no statistically different between our two study groups (40.8 mm^2^ vs. 22.0 mm^2^, *P* = 0.283).

**Conclusions:**

The combination of TBC with CBCT-AF and EBUS is effective in diagnosing PPLs, and a thin cryoprobe is preferred when the PPLs located in difficult areas.

## Introduction

Accurate diagnosis of peripheral pulmonary lesions (PPLs) is an essential step in developing an appropriate treatment plan. Endobronchial ultrasound-guided transbronchial biopsy (EBUS-TBB) is widely used for the diagnosis of PPLs nowadays due to its long history of safety [1–3]. Cone-beam computed tomography-derived augmented fluoroscopy (CBCT-AF) is a technique that provides real-time 2-dimensional (2D) fluoroscopy and 3-dimensional (3D) CBCT scans, and has also been used in bronchoscopy procedures [4–7]. CBCT-AF can guide navigation for the bronchial route, and confirm the location of target PPLs, thereby improving diagnostic accuracy during EBUS-TBB [8, 9]. Traditionally, most TBB procedures employed standard biopsy forceps, which frequently obtained small, crushed tissue samples that could affect molecular analysis for further cancer management [10–12]. Therefore, a better biopsy device to collect larger and higher quality histologic specimens is required.

Cryobiopsy is performed using compressed gas, to create a cooling (Joule-Thomson) effect. This freezes the surrounding tissue, allowing for the extraction of a larger tissue specimen while preserving its internal structure. Transbronchial cryobiopsy (TBC) has become a commonly used method for diagnosing endobronchial tumors and interstitial pulmonary diseases [13–17], and has recently emerged as a diagnostic method for PPLs [18–20]. TBC provides sufficient tissue for gene analysis and histologic subtyping, which helps the physician determine further cancer treatment [11, 21]. Nevertheless, a usual-sized cryoprobe (≥ 1.7 mm) is often cumbersome to use and inflexible, making it difficult to advance to the target bronchus.

The thin cryoprobe (1.1 mm) is more flexible and appears to be easier to operate. However, few studies have reported on the differences between cryoprobes [22, 23]. Furthermore, there is limited evidence regarding the usefulness of CBCT-AF in guiding TBC for the diagnosis of PPLs. Therefore, the aim of this study was to investigate the effectiveness and safety of different-sized cryoprobes in CBCT-AF and EBUS for the diagnosis of PPLs.

## Methods

### Participants

This was a retrospective study of patients who underwent EBUS-TBB and TBC combined with CBCT-AF for the diagnosis of PPLs at the Department of Chest Medicine, National Taiwan University Cancer Center, from January 2021 to May 2022. Patient data, including age, gender, and final diagnosis were collected. To fully characterize the bronchoscopy procedure, the following data were also recorded: the indication (initial diagnosis or re-biopsy), lesion size, lesion pattern (solid or part-solid/ground glass opacity (GGO)), distance from the costal pleura, bronchus sign, location: upper lobes (right upper lobe, and left upper division) or non-upper lobes, visibility on radiograph, position of the EBUS probe (within, adjacent to and invisible the PPLs determined by EBUS image), procedure time, procedure-related major adverse events, radiation dose, intra-procedural CBCT use, TBC failure, and size of the cryoprobe.

Written informed consent was obtained from each patient prior to bronchoscopy. The study received approval from the Institutional Review Board (IRB #202305118RINA) of the National Taiwan University Cancer Center.

### Procedures

All bronchoscopy procedures were performed by our experienced pulmonologists, each with more than eight years of experience in performing bronchoscopy procedures. These procedures were conducted in a hybrid bronchoscopy room equipped with a C-arm CBCT angiography system (Artis Zee Ceiling; Siemens Healthcare GmbH, Forchheim, Germany). The CBCT-AF image was created by the annotation software (syngo iGuide Toolbox; Siemens Healthcare GmbH, Forchheim, Germany) to highlight the area of the target lesion and target bronchus before the bronchoscopy exam.

The patients then underwent conscious sedation with intravenous midazolam, propofol, and fentanyl. A flexible bronchoscope (BF-Q290 or BF-P290; Olympus Co., Tokyo, Japan) was inserted through a supraglottic airway (i-gel®; Intersurgical Ltd, Berkshire, UK), and a 20 MHz radial-EBUS probe (UM-S20-17 S; Olympus Co., Tokyo, Japan) was inserted into the suspected target bronchus, guided by the CT image and navigated via the CBCT-AF image. Intra-procedural CBCT would be performed to confirm EBUS probe within the target lesion if EBUS was unable to identify it due to pure GGO, or atelectasis was suspected. Intra-procedural CBCT would not be performed during the TBB and TBC. After confirming the location of the PPLs, at least six forceps biopsies were performed.

Subsequently, TBC was performed using a flexible cryoprobe (Erbe Elektromedizin GmbH, Tübingen, Germany). Due to the licensing available at our institution, we routinely used a 1.7-mm cryoprobe before August 2021. In September 2021, we started to use a 1.1-mm cryoprobe. The freeze activation time was 3–4 s for the 1.7-mm cryoprobe and 8–10 s for the 1.1-mm cryoprobe. The cryoprobes, which had the frozen tissue specimen at their tip, were removed from the airway along with the bronchoscope as a single unit. The frozen specimen was submerged in saline to rapidly thaw and release it. The bronchoscope was re-inserted into the airway to stop the wound from oozing. The occlusion balloon was not utilized for bleeding prevention. At least two specimens were retrieved via TBC. The severity of bleeding was classified according to the “Delphi Consensus Statement” as follows [24]: Grade 1, which requires suctioning or wedging for ≤ 1 min; Grade 2, suctioning for > 1 min, re-wedging, instillation of cold saline, or vasoactive substances; Grade 3, selective intubation or occlusion balloon for ≤ 20 min; Grade 4, selective intubation for > 20 min, transfer to the intensive care unit (ICU), blood transfusion, or resuscitation.

Rapid on-site cytologic evaluation (ROSE) using a rapid method (Hemacolor; Merck KGaA, Darmstadt, Germany) was always available to confirm lesion access during TBB and TBC. We defined a positive ROSE result as malignant cells on the slide. The lack of malignant cells on the slide revealed a negative ROSE result. We compared ROSE results to the formal pathologic reports on the biopsy specimen. The sensitivity, specificity, positive predictive value (PPV), negative predictive value (NPV), and diagnostic accuracy of ROSE were calculated via standard definitions. All histological specimens obtained through TBB and TBC were finally placed individually in 10% formalin for histological evaluation. The area of the histologic specimen was measured using a digital camera attached to a microscope. Tissue culture and irrigated washing fluid were re-taken for microbiological analysis. The CBCT scan was repeated after completing the bronchoscopy exam to detect any possible pneumothorax. Bronchial brushing, transbronchial needle aspiration, and guide sheath were not used during the procedure.

### Outcomes

The histopathologic diagnostic accuracy was the primary outcome, and we defined the diagnostic accuracy as “correctly biopsy-proved class/total testing class”. The diagnostic criteria for PPLs were established based on cytopathological evidence, microbiological analyses, or clinical follow-up. Histological diagnoses that were inconclusive, such as nonspecific fibrosis, chronic inflammation, or atypia, were deemed non-diagnostic. “Suspicious” findings were also considered as negative in our analysis. Benign inflammations, which could not be determined through cytopathological or microbiological means, were confirmed through radiological and clinical follow-up at least one year after bronchoscopy.

We also evaluated the TBC failure population. The failure of TBC occurred when either we attempted TBC but couldn’t extract a histological specimen, or we didn’t perform TBC because the cryoprobe couldn’t reach the target lesions. To access the possible reasons for the failure of TBC, we also recorded the following data: the location of the lesions (upper lobe position and non-upper lobe position), and the lesions with thick texture after cancer treatment.

### Statistical analysis

We calculated the propensity score using logistic regression. The regression model included indication, location, lesion size, pleural distance, visibility on the radiograph, lesion appearance, probe position, and malignant diagnosis, to reduce the impact of confounding bias. We paired each patient from the 1.7-mm cryoprobe group (1.7 group) with a patient from the 1.1-mm cryoprobe group (1.1 group). To achieve matching, we utilized a caliper width 0.2 times the standard deviation of the propensity score without replacement [25]. After creating the propensity-matched cohort, we conducted an intention-to-treat analysis of the data and calculated the point estimates and 95% confidence intervals (95% CI) of the treatment effect.

After matching, we conducted comparisons using Student’s *t* test or one-way analysis of variance (ANOVA) for continuous variables, and the Chi-squared test or Fisher’s exact test for categorical variables. A significance level of 0.05 was used to determine statistical significance. We utilized SPSS version 22.0 (IBM, SPSS, Chicago, IL) to conduct statistical analysis.

## Results

### Patients

**Fig. 1:**
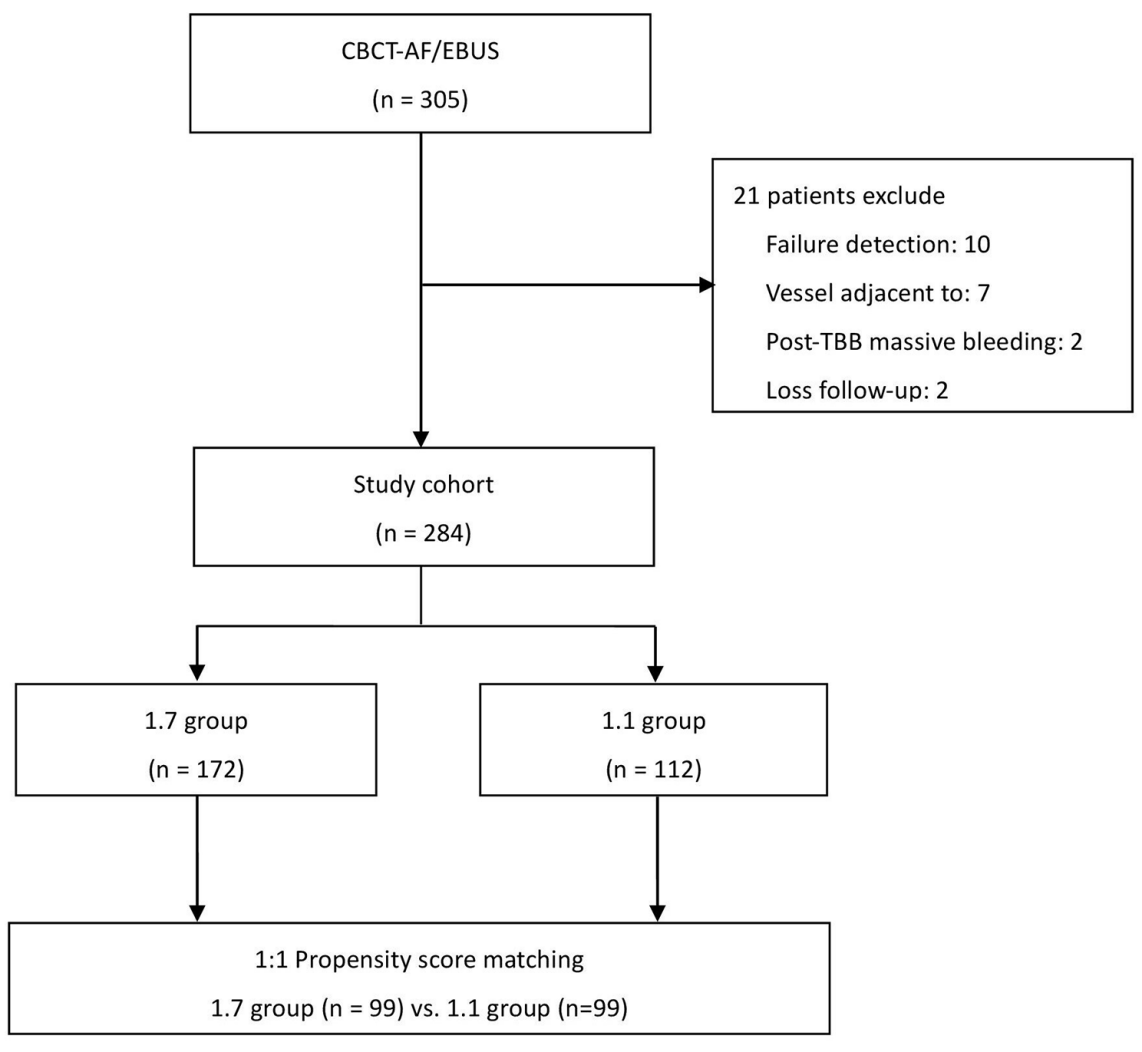
Flowchart for selection of study subjects. CBCT-AF, cone-beam computed tomography-derived augmented fluoroscopy; EBUS, endobronchial ultrasound; n, number

In total, 305 consecutive patients who underwent CBCT-AF and EBUS-TBB for the diagnosis of PPLs were included in our study. Initially, 21 patients were excluded from the study. There were 284 patients, with 172 in the 1.7 group and 112 in the 1.1 group, before propensity score matching. We eventually obtained 99 pairs of patients in both groups. The process for selecting study subjects is outlined in Fig. 1. There were no significant differences in their baseline characteristics or procedure-related complications after matching (Table 1). The final diagnoses for both groups are presented in Table 2.

**Table 1:** Baseline characteristics of patients before and after propensity score matching

Characteristic	Overall cohort (*n* = 284)				Propensity-matched cohort (*n* = 198)		
	1.7 group (*n* = 172)	1.1 group (*n* = 112)	*P*-value		1.7 group (*n* = 99)	1.1 group (*n* = 99)	*P*-value
Age (years old, range)	66.4 (20–91)	65.2 (34–92)	0.540		65.4 (20–91)	66.1 (36–92)	0.533
Male gender (%)	97 (56.4)	50 (44.6)	0.053		55 (55.6)	46 (46.5)	0.201
Scope type							
BF-Q290	83 (48.3)	38 (33.9)	0.012*		32 (32.3)	38 (38.4)	0.229
BF-P290	89 (51.7)	74 (66.1)			67 (67.7)	61 (61.6)	
Lesion character							
Indication (re-biopsy, %)	55 (32.0)	26 (23.2)	0.110		25 (25.3)	26 (26.3)	0.871
Location (%)							
Right upper lobe	45 (26.2)	38 (33.9)	0.102		36 (36.4)	32 (32.3)	0.372
Right middle lobe	22 (12.8)	11 (9.8)	0.445		11 (11.1)	9 (9.1)	0.637
Right lower lobe	45 (26.2)	23 (20.5)	0.173		19 (19.2)	21 (21.2)	0.571
Left upper division	27 (15.7)	24 (21.4)	0.219		20 (20.2)	22 (22.2)	0.728
Left lingual lobe	15 (8.7)	7 (6.3)	0.447		6 (6.1)	7 (7.1)	0.774
Left lower lobe	18 (10.5)	9 (8.0)	0.495		7 (7.1)	8 (8.1)	0.788
Upper lobe (right upper lobe + left upper division)	72 (41.9)	62 (55.4)	0.026*		56 (56.7)	54 (54.5)	0.775
Size (mm, range)	29.1 (8.4–71.3)	24.8 (8-71.1)	0.172		23.5 (8.4–59.4)	25.9 (8.2–71.1)	0.274
Pleural distension (mm, range)	7.3 (0-61.5)	9.4 (0-58.3)	0.236		10.6 (0-61.5)	8.9 (0-58.3)	0.304
Bronchus sign (%)	139 (80.8)	78 (69.6)	0.030*		71 (71.7)	72 (72.7)	0.874
Appearance (%)							
Solid	160 (93.0)	91 (81.3)	0.002*		88 (88.9)	88 (88.9)	1.000
Part solid	7 (4.1)	10 (8.9)	0.052		7 (7.1)	6 (6.1)	0.774
Ground glass opacity	5 (2.9)	11 (9.8)	0.027*		4 (4.0)	5 (5.1)	0.733
Visibility on radiograph (%)	143 (83.1)	85 (75.9)	0.134		71 (71.7)	77 (77.8)	0.326
EBUS probe position (%)							
Within	144 (83.7)	74 (66.1)	0.001*		72 (72.7)	73 (73.7)	0.872
Adjacent to	27 (15.7)	35 (31.25)	0.002*		26 (26.3)	25 (25.3)	0.571
Invisible	1 (0.6)	3 (2.7)	0.171		1 (1.0)	1 (1.0)	0.751
Malignant diagnosis (%)	145 (84.3)	84 (75)	0.053		84 (84.8)	82 (82.8)	0.699
Procedure characteristics							
Intra-procedural CBCT use (%)							
Total case	33 (19.2)	29 (25.9)	0.117		26 (26.3)	21 (21.2)	0.252
Solid case	25/160 (15.6)	14/91 (15.4)	0.556		18/88 (20.5)	14/88 (15.9)	0.186
Part solid case	4/7 (57.1)	7/10 (70)	0.484		4/7 (57.1)	4/6 (66.7)	0.587
Ground glass opacity case	4/5 (80)	8/11 (72.7)	0.635		4/4 (100)	3/5 (60)	0.278
Radiation dose (Gy.cm2)							
Total dose	27.3 (7.6-110.6)	29.2 (7.5-107.2)	0.456		30.1 (10.2-110.6)	27.5 (7.5-105.2)	0.463
Dyna-CT dose	24.5 (6.4–107.0)	26.5 (6.8–103.0)	0.459		27.5 (9.0-106.9)	24.9 (6.8-102.2)	0.459
Fluoroscopy dose	2.6 (0.1–11.8)	2.6 (0.1–12.0)	0.452		2.7 (0.1–11.8)	2.6 (0.1–12.0)	0.478
Procedure duration (minutes, range)	46.7 (20–196)	49.7 (20–183)	0.716		48.7 (20–196)	48.5 (20–183)	0.851
Complications							
Bleeding (%)							
Severe bleeding (Grade 3 + 4)	3 (1.7)	2 (1.8)	0.979		2 (2.0)	2 (2.0)	1.000
Grade 1	63 (36.6)	65 (58.0)			25 (25.3)	58 (58.6)	
Grade 2	80 (46.5)	41 (36.6)			48 (48.5)	38 (38.4)	
Grade 3	2 (1.2)	1 (0.9)			1 (1.0)	1 (1.0)	
Grade 4	1 (0.6)	1 (0.9)			1 (1.0)	1 (1.0)	
Pneumothorax (%)	2 (1.2)	0 (0)	0.252		2 (2.0)	0 (0)	0.155
Fever (%)	5 (2.9)	3 (2.7)	0.909		2 (2.0)	3 (3.0)	0.651
Hypoxia (%)	1 (0.6)	1 (0.9)	0.759		0 (0)	1 (1.0)	0.316
Broken scope (%)	4 (2.3)	0 (0)	0.104		2 (2.0)	0 (0)	0.155

CT, computed tomography; EBUS, endobronchial ultrasound; n, number; *, statistical significance with *P*-value < 0.05.

**Table 2:** Comparison of final diagnoses between the 1.7 group and the 1.1 group

Final diagnosis	1.7 group (*n* = 99)	1.1 group (*n* = 99)
Malignancy (%)	84 (84.8)	82 (82.8)
Lung adenocarcinoma	59 (59.6)	62 (62.6)
Lung squamous cell carcinoma	6 (6.1)	5 (5.1)
Small cell lung cancer	7 (7.1)	2 (2.0)
Lung adenocarcinoma + squamous cell carcinoma	1 (1.0)	0 (0)
Other non-small cell lung cancer	4 (4.0)	7 (7.1)
Lymphoma	0 (0)	1 (1.0)
Invasive thymoma	1 (1.0)	0 (0)
Sarcoma	1 (1.0)	0 (0)
Esophageal cancer	2 (2.0)	0 (0)
Breast cancer	2 (2.0)	1 (1.0)
Hepatocellular carcinoma	1 (1.0)	1 (1.0)
Buccal cancer	0 (0)	1 (1.0)
Prostate cancer	0 (0)	1 (1.0)
Colon cancer	0 (0)	1 (1.0)
Non-malignancy (%)	15 (15.2)	17 (17.2)
Mycobacterium tuberculosis	5 (5.1)	4 (4.0)
Non-tuberculous mycobacteria	0 (0)	3 (3.0)
Bacterial pneumonia	3 (3.0)	0 (0)
Fungal infection	1 (1.0)	2 (2.0)
Aspergillosis	0 (0)	1 (1.0)
Mucomycosis	0 (0)	1 (1.0)
Penicilliosis	1 (1.0)	0 (0)
Bronchiectasis	1 (1.0)	0 (0)
Cryptogenic organizing pneumonia	2 (2.0)	2 (2.0)
Radiation-induced fibrosis	0 (0)	1 (1.0)
Benign inflammation	3 (3.0)	5 (5.1)

n = number.

### Accuracy between different sized cryoprobes

In terms of the overall diagnostic accuracy of TBC, the 1.1-mm cryoprobe was found to be more accurate than the 1.7-mm cryoprobe (80.8% vs. 69.7%, *P* = 0.050). In the subgroup analysis, the 1.1-mm cryoprobe demonstrated higher accuracy when used with PPLs located in the upper lobe (85.2% vs. 66.1%, *P* = 0.020). If the PPLs were smaller than 20 mm, the 1.1-mm cryoprobe had a higher diagnostic accuracy (78.8% vs. 48.8%, *P* = 0.008), and still provided a benefit when the lesion was diagnosed as malignant (90.2% vs. 75%, *P* = 0.010). In addition, the 1.1 group retained a better accuracy when the PPLs were needed to confirm its location under intra-procedural CBCT (42.3% vs. 76.2%, *P* = 0.020) (Table 3).

**Table 3:** Comparison of diagnostic accuracy of 1.7-mm and 1.1-mm cryoprobes

Diagnostic accuracy	1.7 mm cryoprobe (n = 99, %)	1.1 mm cryoprobe (n = 99, %)	*P-*value
Overall	69/99 (69.7)	80/99 (80.8)	0.050*
Initial diagnosis	52/74 (70.3)	58/73 (79.5)	0.200
Re-biopsy	17/25 (68)	22/26 (84.6)	0.162
	*P* = 0.831	*P* = 0.566	
Lesion location			
Upper lobe	37/56 (66.1)	46/54 (85.2)	0.020*
Non-upper lobe	32/43 (74.4)	34/45 (75.6)	0.902
	*P* = 0.370	*P* = 0.226	
Lesion size			
> 20 mm	48/56 (85.7)	54/66 (81.8)	0.562
≤ 20 mm	21/43 (48.8)	26/33 (78.8)	0.008*
	***P*** < 0.001*	*P* = 0.718	
Pleural distension			
≥ 10 mm	34/43 (79.1)	28/32 (87.5)	0.067
< 10 mm	35/56 (62.5)	52/67 (77.6)	0.340
	*P* = 0.075	*P* = 0.243	
Bronchus sign			
Presence	54/71 (76.1)	60/72 (83.3)	0.279
Absence	15/28 (53.6)	20/27 (74.1)	0.114
	***P*** = 0.028*	*P* = 0.297	
Lesion appearance			
Solid	64/88 (72.7)	72/88 (81.8)	0.150
Non-solid	5/11 (45.5)	8/11 (72.7)	0.193
	*P* = 0.064	*P* = 0.470	
Visibility on radiograph			
Visible	51/71 (71.8)	63/77 (81.8)	0.149
Invisible	18/28 (64.3)	17/22 (77.3)	0.320
	*P* = 0.462	*P* = 0.633	
EBUS probe position			
Within	52/72 (72.2)	61/73 (83.6)	0.100
Adjacent to/invisible	17/27 (63.0)	19/26 (73.1)	0.430
	*P* = 0.372	*P* = 0.244	
Intra-procedural CBCT use			
Use	11/26 (42.3)	16/21 (76.2)	0.020*
Not use	58/73 (79.5)	64/78 (82.1)	0.421
	***P*** = 0.001*	*P* = 0.372	
Final diagnosis			
Malignancy	63/84 (75)	74/82 (90.2)	0.010*
Benign	6/15 (40)	6/17 (35.3)	0.784
	***P*** = 0.007*	***P*** < 0.001*	

EBUS, endobronchial ultrasound; n, number; *, statistical significance with *P*-value < 0.05.

In the 1.7 group, the accuracy of TBC also showed a statistically significant difference between lesion sizes (85.7% for sizes > 20 mm vs. 48.8% for sizes ≤ 20 mm, *P* < 0.001) and bronchus sign subgroups (76.1% for presence of a bronchus sign vs. 53.6% for absence of a bronchus sign, *P* = 0.028). Lower accuracy of TBC happened when intra-procedural CBCT needed to be used (42.3% vs. 79.5%, *P* = 0.001). Diagnosis of malignant PPLs had a higher accuracy than that of benign PPLs in both 1.1 and 1.7 subgroups.

### Subgroup analysis of the TBC failure population

More patients had TBC failure in the 1.7 group than in the 1.1 group (20/99, 20.2% vs. 3/99, 3.0%, *P* < 0.001). There was a statistically significant difference between the two groups only in the upper lobe location of the PPLs (15.2% vs. 0%, *P* < 0.001). The detail reasons for TBC failure in the 1.7 group and 1.1 group were showed in Table 4.

**Table 4:** Analysis of the population with TBC failure

	1.7 group (*n* = 99)	1.1 group (*n* = 99)	*P*-value
TBC failure population (n, %)	20 (20.2)	3 (3.0)	< 0.001*
The reason for TBC failure			
Cryoprobe failure to arrive at the lesion	19 (19.2)	1 (1.0)	< 0.001*
Upper lobe position	15 (15.2)	0 (0)	< 0.001*
Non-upper lobe position	4 (4.0)	1 (1.0)	0.174
Lesion too thick	1 (1.0)	2 (2.0)	0.561
Diagnostic accuracy of TBB	15/20 (75)	3/3 (100)	0.328

n, number; TBB, transbronchial biopsy; TBC, transbronchial cryobiopsy; *, statistical significance with *P*-value < 0.05.

After excluding those in the study population who experienced TBC failure, the histologic specimen in both groups was found to be significantly larger with TBC than with TBB (40.8 mm^2^ vs. 7.8 mm^2^, *P* < 0.001 in the 1.7 group; 22.0 mm^2^ vs. 5.3 mm^2^, *P* < 0.001 in the 1.1 group). The mean sample area of the specimen via TBC was not significantly different between the 1.7 and 1.1 groups (40.8 mm^2^ vs. 22.0 mm^2^, *P* = 0.283), nor did the diagnostic accuracy of TBC and TBB show a significant difference between the two groups (Table 5). The ROSE results in both 1.1 and 1.7 groups (175 patients) were showed in Table 6. The sensitivity, specificity, PPV, NPV and diagnostic accuracy of ROSE were 94.9, 89.7, 97.0, 83.3 and 93.7%, respectively.

**Table 5:** Analysis of the population without TBC failure

	1.7 group (*n* = 79)	1.1 group (*n* = 96)	*P*-value
Diagnostic accuracy (n, %)			
TBC	69 (87.3)	80 (83.3)	0.458
TBB	62 (78.5)	77 (80.2)	0.778
	*P* = 0.139	*P* = 0.575	
Area of the histologic specimen (mm^2^, mean, standard deviation)			
TBC	40.8 (± 38.37)	22.0 (± 22.52)	0.283
TBB	7.8 (± 8.13)	5.3 (± 3.67)	0.164
	***P*** < 0.001*	***P*** < 0.001*	

n, number; TBB, transbronchial biopsy; TBC, transbronchial cryobiopsy; *, statistical significance with *P*-value < 0.05.

**Table 6:** Comparison of the results of ROSE with the final pathologic reports by TBC in those without TBC failure (N. = 175)

ROSE	Final Pathologic Reports		Total
	Positive	Negative	
Positive	129	4	133
Negative	7	35	42
Total	136	39	175

Sensitivity = 94.9%, Specificity = 89.7%, Positive predictive value = 97.0%, Negative predictive value = 83.3%, Diagnostic accuracy = 93.7%. N. = number; ROSE = rapid on-site cytologic evaluation; TBC = transbronchial cryobiopsy.

## Discussion

This retrospective study found that TBC guided by CBCT-AF and EBUS had comparable diagnostic accuracy to TBB, with larger tissue specimens. When comparing cryoprobes of different sizes, the use of a 1.1-mm cryoprobe resulted in better diagnostic accuracy than a 1.7-mm cryoprobe, particularly for PPLs located in the upper lobe, small lesion sizes, and intra-procedural CBCT was needed to be used. The size of the tissue specimens did not show a statistically significant difference between the 1.7 and 1.1 groups.

To achieve a consensus on PPLs diagnosis, at least three major steps: navigation, confirmation, and acquisition, are required to implement the transbronchial procedure [4, 5]. In this study, we utilized CBCT-AF for precise and nearly real-time navigation, radial-EBUS to confirm target lesions, and ROSE to immediately affirm the adequacy of the diagnostic samples acquired. Diagnostic accuracy with TBB was 78.5% in the 1.7 group and 80.2% in the 1.1 group. This confirms our previous results that using CBCT-AF, ROSE, and EBUS simultaneously has a relatively high accuracy for TBB diagnosis [9]. There was no statistically significant difference in accuracy between TBC and TBB in both groups after excluding TBC failure population. In addition, the tissue specimen was significantly larger with TBC than with TBB in both of our study groups. Previous literature has reported that tissue specimens obtained via cryobiopsy are significantly larger than those obtained via forceps biopsy, resulting in a higher detection rate for gene analysis [11]. Therefore, the combination of TBC with EBUS and CBCT-AF is an effective method for diagnosing PPLs.

The acquisition of larger specimens can lead to greater destruction of lung parenchyma, thereby increasing the risk of bleeding and pneumothorax [14, 26]. The cryoprobe has to be removed together with the endoscope during TBC procedure. This necessitates re-insertion of the bronchoscope might also raise the risk of severe bleeding. In the present study, the rate of severe bleeding (grade 3 and grade 4) was approximately 2% in both the 1.7 and 1.1 groups. One patient in each group experienced grade 4 bleeding, requiring transfer to the ICU for monitoring. Two patients in the 1.7 group suffered from mild pneumothorax and did not require further drainage. Severe bleeding and pneumothorax were uncommon in our study population, and this may be attributed to the routine combination of radial-EBUS and CBCT-AF in all procedures. Radial-EBUS can detect vessels within PPLs, thereby decreasing the risk of severe bleeding by avoiding injury to large vessels [27, 28]. CBCT-AF can provide real-time imaging and help prevent pleural injury [5, 9]. Previous literature has also reported that severe bleeding and pneumothorax rarely occurred after TBC [20, 29]. Though serious complication causing by TBC still require vigilance, the incidence could be minimized and more controllable when CBCT-AF and radial-EBUS are used together.

However, performing TBC in strongly bent bronchi, such as the upper lobe bronchus or distal small airway, is challenging due to the rigidity of usual-sized cryoprobes [30]. This can lead to difficulties in accurately targeting lesions that are located in the upper lobe, close to the pleura, non-solid pattern, small size, negative bronchus sign, or invisible in radiographs. Intra-procedural CBCT might also be required to confirm the location of the target PPLs in these difficultly diagnosed conditions. As a result, cryoprobe may have difficulty approaching the lesion, leading to lower accuracy rates [31–34]. In the 1.7 group, smaller lesion sizes, the absence of bronchus signs, and intra-procedural CBCT used were associated with lower TBC accuracy rates. Nineteen patients (19.2%) could not undergo TBC due to cryoprobe failure upon arrival at the PPLs. The majority of these patients (15/19, 78.9%) had upper lobe locations. There was also a trend of decreasing accuracy in TBC diagnosis when PPLs were located in the upper lobe, had pleural distension shorter than 10 mm, had non-solid appearance, or were invisible in radiographs. Furthermore, four cases experienced scope injury with working channel leakage before propensity score matching, and two cases after propensity score matching following the procedures. All of the injuries occurred due to a reluctance to advance to the upper lobe PPLs, even though TBC was successfully performed. These situations indicated that a cryoprobe of usual size is too bulky to access PPLs in difficult areas.

The thin cryoprobe has a small outer diameter and is easily bendable, making it easier to expand to a farther range than usual-sized cryoprobes [29]. As a result, the failure rate of the cryoprobe reaching the target PPLs was significantly lower in the 1.1 group than in the 1.7 group (1.0% vs. 19.2%, *P* < 0.001). The thin cryoprobe demonstrated higher diagnostic accuracy not only in the overall population but also in PPLs with small lesion size, upper lobe location, and intra-procedural CBCT used. Although there was no statistical significance, there was still a trend towards increased accuracy in TBC when using a 1.1-mm cryoprobe with shorter pleural distension, non-solid patterns and absence of bronchus sign. In addition, the diagnostic accuracy of the factors, which we mentioned above were no significant differences in the 1.1 group. This result is similar to Kim’s report [35]. No episode of scope injury was also observed in the 1.1 group. Using a thin cryoprobe appears to make TBC easier to perform.

A thin cryoprobe would obtain a smaller sample than a standard-sized cryoprobe during an endobronchial biopsy. To compensate the situation, we used longer freeze activation time in the 1.1 group than in the 1.7 group. There is still a trend of larger sample size obtained in 1.7 group, but there is no statistically different between our two study groups (40.8 mm^2^ vs. 22.0 mm^2^, *P* = 0.283). In addition, the tissue specimen was significantly larger with TBC than with TBB in both of our study groups. We believed the use of a thin cryoprobe with appropriate freeze time appears to be effective in obtaining sufficient tissue during TBC procedures.

Apart from the failure of the cryoprobe to arrive during the lesion approach, another reason for failure in our study was the presence of PPLs with a thick texture. Tumor fibrosis may occur after cancer treatment [36, 37], resulting in a thicker texture that makes it more difficult to extract tissue specimens during re-biopsy. It is more difficult to extract large specimens via TBC than small specimens via TBB because more strength is needed to break through the surrounding fibrotic tissue. In our study, one patient in the 1.7 group and two patients in the 1.1 group were unable to undergo the procedure due to lesions with thick textures. However, all of these patients were successfully diagnosed through forceps biopsy. We also found that the accuracy of TBB remained acceptable in the TBC failure population, with a success rate of 75% in the 1.7 group and 100% in the 1.1 group. TBC cannot completely replace TBB in these situations.

Some study revealed that the diagnostic accuracy of re-biopsy would be decreased because of tumor fibrosis and necrosis occurred after cancer treatment [36, 37]. This might increase the heterogenous in the study population; therefore, many studies excluded the re-biopsy population. To minimize the impact, we used indication (initial diagnosis vs. re-biopsy) as one factor for matching and the proportion of re-biopsy population was no different in the two study groups. In addition, the accuracy was also similar between the population for initial diagnosis or for re-biopsy. The reason is that ROSE with high accuracy helps immediate feedback on the quality of the biopsy sample, thereby confirms the adequate biopsy site [3]. We believe our study population is more similar in the real world, and our results can be applied to the clinical practice.

Our study has several limitations. First, this was a retrospective study. Although we used propensity score matching to minimize the bias, we could not account for the potential confounding factors. Positive findings that are actually meaningful could also have been missed. It is also difficult for us to standardize the protocol of TBC procedure in all our study population. Some factors, such as the use of different types of bronchoscopes, and the use of intra-procedural CBCT might also influence the diagnostic accuracy of TBC. However, we found that there was no statistic difference after the propensity score matching. We believe the effect might be minimized in this situation and we can still apply our results in the clinical practice. Second, this was a single-center study, and our study population was relatively homogeneous. More than 80% of the study population received a final diagnosis of malignancy. We know that the accuracy of diagnosing malignant PPLs is actually higher than that of benign processes [38]. However, there is a risk that this may not be generalizable to patients in other institutions. Therefore, a prospective randomized study with a comprehensive study group is warranted.

Third, the introduction of the 1.1-mm cryoprobe in our institution came later than the introduction of the 1.7-mm cryoprobe. It is inappropriate for us to ignore the possibility that the technical familiarity with the use of the 1.1-mm cryoprobe might boost the diagnostic performance of the 1.7-mm cryoprobe. In addition, the diagnostic accuracy of TBC in Tanaka’s study was relatively high, and all 1.7-mm cryoprobe could be advanced to the PPLs located upper lobe [39]. All operators in the study were experts with at least 100 cases of experience performing cryobiopsy using conventional cryoprobes (1.9-mm cryoprobe). We believe the experience of the operator might also influence the success rate of TBC. Forth, all patients underwent TBB followed by TBC in a single procedure; therefore, it was difficult to distinguish between the complications of TBB and those of TBC. In addition, one study supposed that initial TBB might alter the surrounding environments of PLLs, such as the formation of organized blood clots or an edematous change of the target bronchus, thus reducing the accuracy of TBC [40]. Though the situation was not reported in other studies, the possible impact remained to be cautious. Unlike other studies, we did not exclude individuals who attempted TBC but ultimately failed. Although this may decrease the diagnostic accuracy, we can still observe the challenges of TBC in the real world.

In conclusion, combining TBC with CBCT-AF and EBUS is an effective method for diagnosing PPLs, though severe complication is cautionary but controllable. This approach provides valid accuracy, and allows for obtaining larger tissue specimens. The thin cryoprobe is more flexible, which improves the accuracy of diagnosing PPLs with small lesion sizes and difficult locations. The histologic sample size obtained through a thin cryoprobe is not inferior to that obtained through a usual-sized cryoprobe. We believe that the combination of CBCT-AF and EBUS, along with TBC using a thin cryoprobe, will be valuable tools in the era of gene-guided therapy. However, conventional biopsy devices cannot be abandoned when TBC fails to accomplish its purpose.

## Data Availability

No datasets were generated or analysed during the current study.

## Abbreviations

2D: 2-dimensional
3D: 3-dimensional
CBCT-AF: cone-beam computed tomography-derived augmented fluoroscopy
CT: computed tomography
EBUS: endobronchial ultrasound
EBUS-TBB: endobronchial ultrasound-guided transbronchial biopsy
GGO: ground glass opacity
ICU: intensive care unit
NPV: negative predictive value
PPLs: peripheral pulmonary lesions
PPV: positive predictive value
ROSE: rapid on-site cytologic evaluation
TBB: transbronchial biopsy
TBC: transbronchial cryobiopsy
